# Four New Species of *Deconica* (Strophariaceae, Agaricales) from Subtropical Regions of China

**DOI:** 10.3390/jof10110745

**Published:** 2024-10-29

**Authors:** Jun-Qing Yan, Sheng-Nan Wang, Ya-Ping Hu, Cheng-Feng Nie, Bin-Rong Ke, Zhi-Heng Zeng, Hui Zeng

**Affiliations:** 1 Institute of Edible mushroom, Fujian Academy of Agricultural Sciences, Fuzhou 350011, China; yanjunqing1990@126.com (J.-Q.Y.); niechenfeng6@gmail.com (C.-F.N.); kebinrong@163.com (B.-R.K.); 2 Jiangxi Key Laboratory of Subtropical Forest Resourecs Cultivation, Jiangxi Agricultural University, Nanchang 330045, China; wangshengnan_2003@163.com; 3 Nanjing Institute of Environmental Sciences, Ministry of Ecology and Environment Mountains, Nanjing 210042, China; huyap9009@163.com

**Keywords:** basidiomycetes, four new taxa, taxonomy, phylogeny

## Abstract

*Deconica* is a relatively small genus, with only 90 names recorded in previous research. In this study, four new species of *Deconica* have been identified based on morphological and phylogenetic evidence from subtropical regions of China. This represents the first discovery of new species of *Deconica* in China. Morphologically, *D*. *austrosinensis* is characterized by medium-sized spores that are elliptical to elongated-ellipsoid in face view, and fusiform to sublageniform and slightly thick-walled pleurocystidia; *D*. *furfuracea* is identified by a well-developed and evanescent veil, medium-sized spores that are rhomboid to mitriform in face view, and fusiform to subclavate pleurocystidia that are rare and subacute at apex; *D*. *fuscobrunnea* is recognized by dark brown pileus, medium-sized spores that are rhomboid to mitriform in face view, an ixocutis pileipellis, lageniform cheilocystidia with a long neck and lacks pleurocystidia; *D*. *ovispora* is distinguished from other *Deconica* species by medium-sized spores that are ovoid in face view, an ixocutis pileipellis, lageniform cheilocystidia with a long to short neck, and lacks pleurocystidia. Their distinct taxonomic status is confirmed by the positions of the four new species in ITS + LSU phylogenetic trees. Detailed descriptions and morphological photographs of four new species are presented.

## 1. Introduction

*Deconica* (W.G. Sm.) P. Karst. is characterized by usually mycenoid, collybioid, omphaloid, or crepidotoid basidiomata; conical, convex or hemispherical, dry or viscid pileus with or without an umbo; brown to purple-brown, adnate to broadly adnate lamellae with a decurrent tooth in most species; and a slender stipe when present; they often grow on rotten wood, grasses, mosses, trunks, and dung [1,2]. For a long time, all species of *Deconica* were considered part of *Psilocybe* (Fr.) P. Kumm until phylogenetic studies were conducted [3]. Morphologically, *Deconica* differ from *Psilocybe* by the absence of hallucinogenic compounds, the broadly attached lamellae with a decurrent tooth in most species, and never blackish purple lamellae [1,2].

*Deconica* is not a very species-rich genus: only 90 names (55 species and 2 varieties), including synonyms and subspecies, were listed in Index Fungorum, since Smith established the subgenus *Deconica* under *Agaricus* L. [4,5]. Based on the study of Guzmán, there could be around 133 species of *Deconica* worldwide [6]. Noordeloos pointed out that there are 24 species in Europe and Ramírez-Cruz treated 47 taxa worldwide [1,2,7]. In previous studies, only six species of *Deconica* were reported in China [8,9]. As part of the study on Chinese macrofungi species, four new species were discovered, during our investigations in subtropical regions of China, and this also marks the first discovery of new species of this genus in China. In this paper, detailed information on the new taxa is presented.

## 2. Materials and Methods

### 2.1. Morphological Studies

Specimens were collected from Fujian, Hubei, Jiangxi, and Zhejiang provinces of China between 2019 and 2024 and were deposited in the Herbarium of Fungi, Jiangxi Agricultural University (HFJAU). Macroscopic descriptions were based on detailed field notes of fresh basidiomata and photos. Colour codes follow the Methuen Handbook of Colour [10]. Microscopic structures were observed and measured from dried specimens mounted in water, 5% KOH. Congo red was used as a stain when necessary [11]. Patent Blue V 0.1% was used to detect chrysocystidia [12]. At least 20 basidiospores, basidia, and cystidia were measured for each collection. The range of spore size is expressed as the form (a) b–c (d), in which “a” and “d” represent the minimum and maximum values, 90% of the spores fall within the range ‘b–c’. The meanings of the other spore characteristics are as follows: “Q” stands for the ratio of length and width [13,14].

### 2.2. DNA Extraction, PCR Amplification, and Sequencing

DNA was extracted from dried specimens with the NuClean Plant Genomic DNA kit (CWBIO, China). Two regions (ITS, LSU) were selected for the study and were amplified using the primer pairs ITS1/ITS4 [15], LR0R/LR7 [16,17], respectively. PCR was performed using a touchdown program for all regions: initial 95 °C for 5 min, and then 14 cycles of denaturing at 95 °C for 30 s, annealing at 65 °C for 45 s (−1 °C per cycle), extension at 72 °C for 1 min; then 30 cycles of denaturing at 95 °C for 30 s, annealing at 52 °C for 30 s, extension at 72 °C for 1 min; final extension at 72 °C for 10 min [13,18]. The PCR products were sequenced by Qing Ke Biotechnology Co., Ltd. (Wuhan, China).

### 2.3. Alignment and Phylogenetic Analyses

Sequence reads were assembled and edited using Sequencher v.5.4 and were deposited in GenBank (GB) database. In total, 32 sequences, including ITS and LSU regions, were generated from our collected specimens. Based on the research by Ramírez-Cruz and colleagues [1,17], and the similarity of these new species to the most closely related sequences identified in the BLAST results of ITS, 85 nucleotide DNA sequences in NCBI GenBank were downloaded. The *Kuehneromyces brunneoalbescens* (Y.S. Chang & A.K. Mills) J.A. Cooper was chosen as an outgroup taxon according to the results of Ramírez-Cruz and colleagues [1]. Details are presented in Table 1.

**Figure 1:**
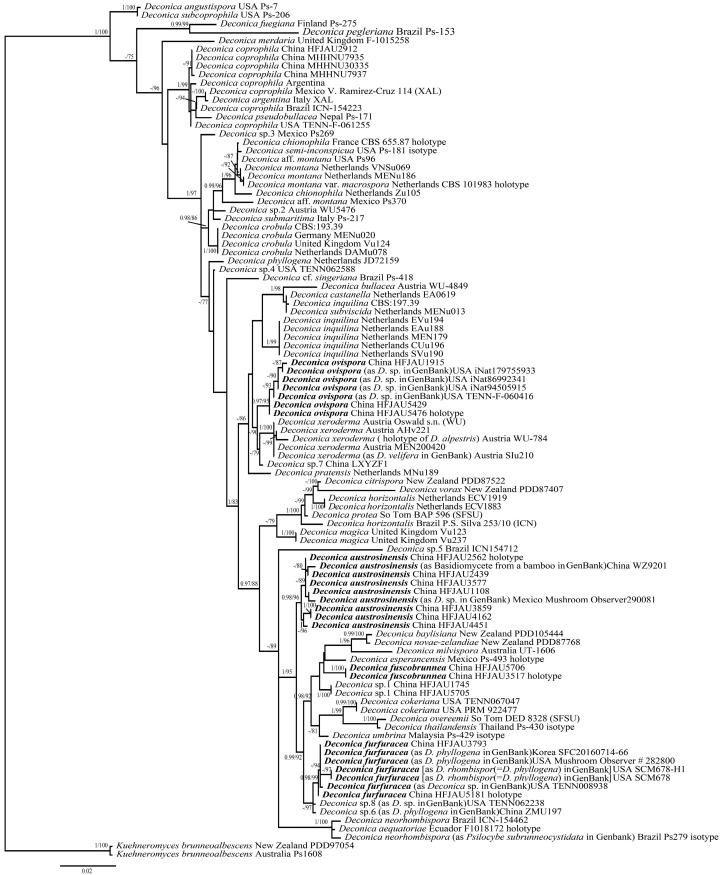
Phylogram of *Deconica* spp. generated with maximum likelihood (ML) analysis based on ITS and LSU, rooted with *Kuehneromyces brunneoalbescens*. Bayesian inference (BI-PP) ≥ 0.95 and ML bootstrap proportions (ML-BP) ≥ 75 are indicated as PP/BP. The new taxa are marked in bold.

ITS and LSU sequence datasets were separately aligned on the MAFFT v.7 [19]. Bayesian inference (BI) and maximum likelihood (ML) phylogenetic analyses of the aligned concatenated dataset were respectively carried out in MrBayes v.3.2.7a and IQtree v.2.1.2, respectively [20,21]. The best-fit models of BI were determined by PartitionFinder, complying with the Corrected Akaike information criterion (AICc) [22]. The ML analysis was conducted using the ultrafast bootstrap option with 1000 replicates and allowing partitions to have different seeds (--p). For the BI analysis, four Monte Carlo Markov chains were run for 5 million generations, sampling every 100th generation, with the first 25% of trees discarded as burn-in. The branches of Bayesian posterior probability (BI-PP) ≥ 0.95 and ML bootstrap support (ML-BP) ≥ 75% are considered statistical supports and are shown in the tree (Figure 1). All alignments for phylogenetic analyses and tree were deposited in TreeBASE (ID: TB2:S31681).

**Table 1:** Details of sequences used in the phylogenetic analyses.

Species	Voucher	Country	ITS	LSU	References
*D*. *aequatoriae*	F1018172 holotype	Ecuador	MT622203		[1]
*D*. *angustispora*	Ps-7	USA	MT622205		[1]
*D*. *argentina*	XAL	Italy	KC669307		[23]
*D*. *austrosinensis*	HFJAU2439	China	PP759373	PQ282655	This study
*D*. *austrosinensis*	HFJAU2562 holotype	China	PP759375	PQ282656	This study
*D*. *austrosinensis*	HFJAU3577	China	PP759374	PQ282659	This study
*D*. *austrosinensis*	HFJAU3859	China	PQ282646	PQ282661	This study
*D*. *austrosinensis*	HFJAU4162	China	PQ282647	PQ282662	This study
*D*. *austrosinensis*	HFJAU4451	China	PQ282648	PQ282663	This study
*D*. *austrosinensis*	HFJAU1108	China	MN622718		This study
*D. austrosinensis* (as Basidiomycete from a bamboo in GB)	WZ9201	China	U65602		[24]
*D. austrosinensis* (as *D*. sp. in GB)	Mushroom Observer290081	Mexico	MH159224		Unpublished
*D. baylisiana*	PDD105444	New Zealand	KM975393		[23]
*D*. *bullacea*	WU-4849	Austria	MT622207		[1]
*D*. *castanella*	EA0619	Netherlands	MT622208		[1]
*D*. *chionophila*	CBS 655.87 holotype	France	MH862111	NG_068969	[3]
*D*. *chionophila*	Zu105	Netherlands	MT622209		[1]
*D*. *citrispora*	PDD87522	New Zealand	KM975431		[23]
*D*. *cokeriana*	TENN–F–067047	USA	KC669315		[23]
*D*. *cokeriana*	PRM 922477	USA	MK965914		Unpublished
*D*. *coprophila*	HFJAU2912	China	PQ282645	PQ282657	This study
*D*. *coprophila*	V. Ramirez-Cruz 114	Mexico	KC669308	KC669336	[17]
*D*. *coprophila*		Argentina	JX235960		[25]
*D*. *coprophila*	MHHNU30335	China	MK214386		Unpublished
*D. coprophila*	ICN-154223	Brazil	MT622210		[1]
*D. coprophila*	TENN–F–061255	USA	MT622211		[1]
*D*. *coprophila*	MHHNU7935	China	OP862790		Unpublished
*D*. *coprophila*	MHHNU7937	China	OP862791		Unpublished
*D*. *crobula*	CBS:193.39		MH855980		[26]
*D*. *crobula*	DAMu078	Netherlands	MT622213		[1]
*D*. *crobula*	MENu020	Germany	MT622214		[1]
*D*. *crobula*	Vu124	UK	MT622215		[1]
*D*. *esperancensis*	Ps-493 holotype	Mexico	MT622216		[1]
*D*. *fuegiana*	Ps-275	Finland	MT622217		[1]
*D. furfuracea*	HFJAU3793	China	PP759377	PQ282660	This study
*D. furfuracea*	HFJAU5181 holotype	China	PP759378	PQ282664	This study
*D. furfuracea* (as *D. phyllogena* in GB)	SFC20160714-66	Korea	MF437002		[27]
*D. furfuracea* (as *D. phyllogena* in GB)	Mushroom Observer # 282800	USA	MK607529		Unpublished
*D. furfuracea* [as *D. rhombispor* (=*D. phyllogena*) in GB]	SCM678-H1	USA	FJ596920		[28]
*D. furfuracea* [as *D. rhombispor* (=*D. phyllogena*) in GB]	SCM678	USA	FJ596921		[28]
*D. furfuracea* (as *D.* sp. in GB)	TENN–F–008938	USA	MT622256		[1]
*D. fuscobrunnea*	HFJAU3517 holotype	China	PP759379	PQ282658	This study
*D. fuscobrunnea*	HFJAU5706	China	PQ282652		This study
*D*. *horizontalis*	P.S. Silva 253/10 (ICN)	Brazil	KC669309	KC669337	[17]
*D*. *horizontalis*	ECV1919	Netherlands	MT622219		[1]
*D*. *horizontalis*	ECV1883	Netherlands	MT622220		[1]
*D*. *inquilina*	CBS:197.39		MH855982		Unpublished
*D*. *inquilina*	EVu194	Netherlands	MT622221		[1]
*D*. *inquilina*	CUu196	Netherlands	MT622222		[1]
*D*. *inquilina*	MEN179	Netherlands	MT622223		[1]
*D*. *inquilina*	EAu188	Netherlands	MT622224		[1]
*D*. *inquilina*	SVu190	Netherlands	MT622225		[1]
*D*. *magica*	Vu123	UK	MT622226		[1]
*D*. *magica*	Vu237	UK	MT622227		[1]
*D*. *merdaria*	F-1015258	UK	MT622228		[1]
*D*. *milvispora*	UT-1606	Australia	KC669314	NG_081284	[1]
*D*. *montana*	VNSu069	Netherlands	MT622231		[1]
*D*. *montana*	MENu186	Netherlands	MT622232		[1]
*D*. *montana* var. *macrospora*	CBS 101983 holotype	Netherlands	MH862774	MH874369	[26]
*D*. aff. *montana*	Ps–370	Mexico	KC669311		[1]
*D*. aff. *montana*	Ps–96	USA	MT622229		[1]
*D*. *neorhombispora*	ICN-154462	Brazil	MT622234		[1]
*D. neorhombispora* (as *P. subrunneocystidata* in GB)	Ps–279 isotype	Brazil	MT622233		[1]
*D*. *novae*-*zelandiae*	PDD87768	New Zealand	KM975401		[1]
*D*. *overeemii*	DED 8328 (SFSU)	São Tomé	KX017212		[29]
*D. ovispora*	HFJAU1915	China	PQ282644	PQ282654	This study
*D. ovispora*	HFJAU5429	China	PQ282649		This study
*D. ovispora*	HFJAU5476 holotype	China	PQ282650		This study
*D. ovispora* (as *D.* sp. in GB)	TENN–F–060416	USA	MT622259		[1]
*D. ovispora* (as *D*. sp. in GB)	iNat86992341	USA	ON774784		Unpublished
*D. ovispora* (as *D.* sp. in GB)	iNat94505915	USA	OP270521		Unpublished
*D. ovispora* (as *D. sp.* in GB)	iNat179755933	USA	OR825659		Unpublished
*D*. *pegleriana*	Ps-153	Brazil	MT622235		[1]
*D*. *phyllogena*	JD72159	Netherlands	MT622236		[1]
*D*. *pratensis*	MNu189	Netherlands	MT622238		[1]
*D*. *protea*	BAP 596 (SFSU)	São Tomé	KX017213		[29]
*D*. *pseudobullacea*	Ps-171	Nepal	MT622239		[1]
*D*. *semi*-*inconspicua*	Ps-181 isotype	USA	MT622240		[1]
*D*. cf. *singeriana*	Ps-418	Brazil	MT622241		[1]
*D*. *subcoprophila*	Ps-206	USA	MT622242		[1]
*D*. *submaritima*	Ps-217	Italy	MT622243		[1]
*D*. *subviscida*	MENu013	Netherlands	MT622244		[1]
*D*. *thailandensis*	Ps-430 isotype	Thailand	MT622245		[1]
*D*. *umbrina*	Ps-429 isotype	Malaysia	MT622246		[1]
*D*. *vorax*	PDD87407	New Zealand	KM975441		[1]
*D*. *xeroderma*	Oswald s.n. (WU)	Austria	KC669312	KC669340	[17]
*D*. *xeroderma*	AHv221	Austria	MT622248		[1]
*D*. *xeroderma*	MEN200420	Austria	MT622249		[1]
*D*. *xeroderma* (type of *D*. *alpestris*)	WU-784 holotype	Austria	MT622204		[1]
*D*. *xeroderma* (as *D*. *velifera* in GB)	SIu210	Austria	MT622247		[1]
*D*. sp.1	HFJAU1745	China	PQ282643	PQ282653	This study
*D*. sp.1	HFJAU5705	China	PQ282651		This study
*D*. sp.2	WU5476	Austria	MT622261		[1]
*D*. sp.3	Ps–269	Mexico	MT622265		[1]
*D*. sp.4	TENN–F–062588	USA	KC669316		[1]
*D*. sp.5	ICN–154712	Brazil	MT622254		[1]
*D*. sp.6 (as *D*. *phyllogena* in GB)	ZMU197	China	MW724279		Unpublished
*D*. sp.7	LXYZF1	China	MZ452395		[1]
*D*. sp.8	TENN–F–062238	USA	KC669313		[1]
*Kuehneromyces brunneoalbescens*	PDD97054	New Zealand	KM975426	KM975380	[23]
*K. brunneoalbescens*	Ps1608	Australia	MK965912		[1]

## 3. Results

### 3.1. Phylogenetic Analysis

A total of 1798 characters from 101 taxa were used in phylogenetic analyses (ITS, 624 bp; LSU, 1174 bp), of which 227 sites were variable and 163 were parsimony informative for ITS, and 56 sites were variable and 34 were parsimony informative for LSU. Due to the different number of models supported by Mrbayes and IQtree, the best models are calculated separately, and the results are as follows: the best models for Bayesian analysis were GTR + I + G for the ITS, HKY + I + G for the LSU; the best models for ML analysis were: TPM2u + F + I + G4 for the ITS, K2P + I + G4 for the LSU. For the Bayes analysis, the average standard deviation of split frequencies was less than 0.01 after 1.57 million generations.

As shown in the ML tree in Figure 1, four new species formed distinct and stable branches, respectively. *D. furfuracea* groups together with two unknown species (*D.* sp.8 and *D.* sp.6), but this grouping has unstable support in Bayesian analysis. Similarly, *D. ovispora* groups together with *D. xeroderma*, and this grouping also has unstable support in Bayesian analysis.

### 3.2. Taxonomy

**Figure 2:**
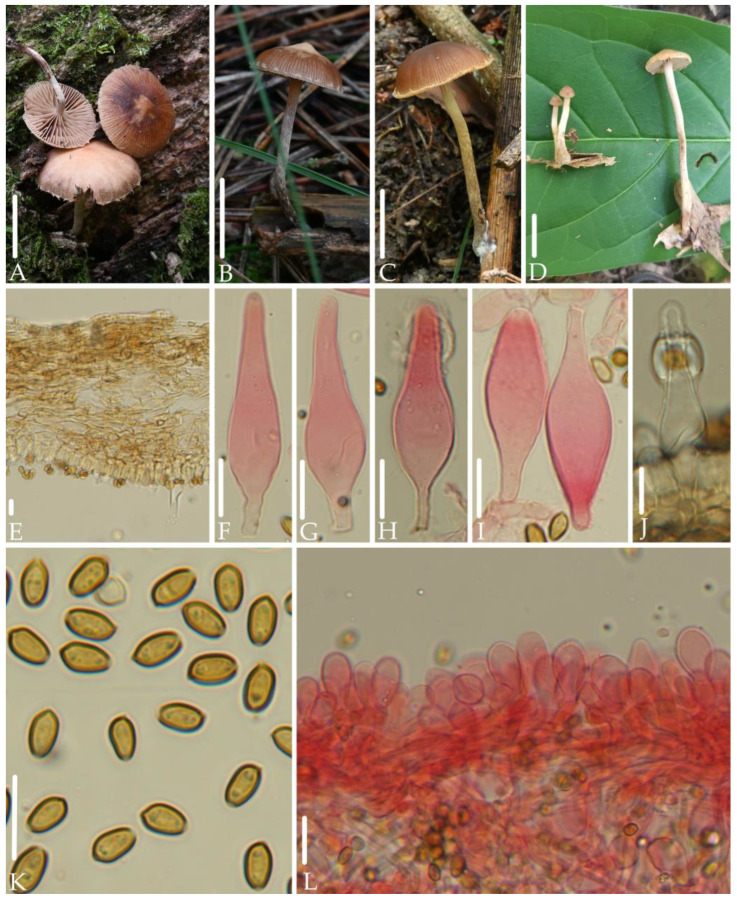
Morphological structures of *Deconica austrosinensis.* (**A**–**D**) Basidiomata. (**E**) Pileipellis. (**F**–**J**) Pleurocystidia. (**K**) Spores. (**L**) Cheilocystidia. Scale bars: (**A**–**D**) 10 mm, (**E**–**L**) 10 μm. All microstructures were observed in 5%KOH. Structures of (**F**–**I**,**L**) were stained with 1%Congo red.

*Deconica austrosinensis* J.Q. Yan, S.N. Wang, and H. Zeng sp. nov. (Figure 2).

MycoBank: 855638

*Etymology*. “austrosinensis” refers to its type specimen originating from the southern regions of China.

*Holotype*. China, Fujian Province, Wuyishan National Park, 18 June 2021, collected by Jun-Qing Yan, and Sheng-Nan Wang, HFJAU2562.

*Diagnosis*. *Deconica austrosinensis* is mainly characterized by very small basidiomata; hygrophanous, reddish brown, striate pileus; ellipsoid to elongated-ellipsoid spores in face view, (5.4) 5.8–7.0 × 3.7–4.5 (4.8) µm; fusiform to sublageniform, slightly thick-walled pleurocystidia; clavate to pyriform, rarely fusiform, thin-walled cheilocystidia. It differs from *D. cokeriana* by clavate to pyriform, rarely fusiform cheilocystidia, and lack of chrysocystidia.

*Description*. Basidiomata very small. Pileus 11–20 mm, plano-convex to plane, rarely with unobviously obtuse umbo, hygrophanous, reddish brown (8D5–8E5), striate up to center from the margin, becoming grayish red (8C5) to dull red (8B4–8B5) as drying. Veil dull red (8B4–8B5), scattered or only remains on the edge of the pileus, fibrillose, evanescent. Context thin, 1.0 mm at the center. Lamellae 1.5–2.0 mm, decurrent to short decurrent, distant, unequal, dull red (8B4–8B5), edge even to slightly serrate, white. Stipe 10–30 mm long, 1.5–2.0 mm thick, central, cylindric, equal, dull red (8B4–8B5), gradually darkening toward the base, covered with white and evanescent fibrillose.

Spores (5.4) 5.8–7.0 × 3.7–4.5 (4.8) µm, Q = (1.3) 1.4–1.8, ellipsoid to elongated-ellipsoid in face view, 3.5–4.0 (4.4) µm broad, elongated-ellipsoid in profile, slightly thick-walled, smooth, brownish-yellow, germ pore distinct, 0.8–1.5 µm broad. Basidia 13–20 (23) × (5.0) 5.5–7.2 µm, subcylindric to clavate, pale yellow to hyaline, 4-spored. Pleurocystidia (30) 35–53 (56) × (8.0) 9.0–13 (16) µm, sublageniform with or without amorphous deposits at apex, fusiform with or without mucronate, slightly thick-walled, hyaline. Cheilocystidia 15–30 × (5.0) 7.4–12 µm, clavate to pyriform, rarely fusiform, thin-walled, hyaline. Pileipellis a cutis, hyphae 3.0–7.0 µm broad, pale yellow to brown-yellow. Clamp connections present.

*Habitat.* Solitary to scattered on rotten wood or humus in broad-leaved forests.

*Additional specimens examined*: China, Fujian Province, Wuyishan National Park, 18 June 2021, collected by Jun-Qing Yan, and Sheng-Nan Wang, HFJAU2439, HFJAU2562; 7 June 2022, collected by Bin-Rong Ke and Ya-Ping Hu, HFJAU3577; 27 June 2022, collected by Bin-Rong Ke and Cheng-Feng Nie, HFJAU3859, HFJAU3906; 8 July 2022, collected by Zhi-Heng Zeng, Hui Zeng HFJAU4451; Jiangxi Province, Lushan National Nature Reserve, collected by Jun-Qing Yan,10 July 2019, HFJAU1108; Zhejiang Province, Qingtian County, Lishui City, 8 August 2021, collected by Jun-Qing Yan, HFJAU2971.

*Notes*. This species forms an independent branch in the phylogenetic tree, showing clear genetic differentiation from other known *Deconica* spp. Morphologically, among the known species of the *Deconica*, few have a similar combination of characteristics as *D. austrosinensis*, that is, spores that are elliptical to elongated-ellipsoid in face view, with a length concentrated between 6.0–7.0 μm, and present of pleurocystidia. However, they can be clearly distinguished from *D. austrosinensis*: The pleurocystidia of *D. hartii* (Ammirati) Ammirati & Redhead are cylindrical to ventricose, and shorter than 25 μm [30]; *D. cokeriana* (A.H. Sm. & Hesler) Ram.-Cruz & A. Cortés-Pérez has chrysocystidia that are thin-walled and shorter than 40 μm, and its cheilocystidia are lageniform to widely utriform [23].

In addition, *D. austrosinensis* forms a clade with two unknown sequences from Mexico (MH159224) and Sichuan Province, China (U65602), and shares over 99% ITS similarity with them. It is highly likely that these two unknown sequences represent the distribution of the species in Mexico and Yunnan Province, China.

**Figure 3:**
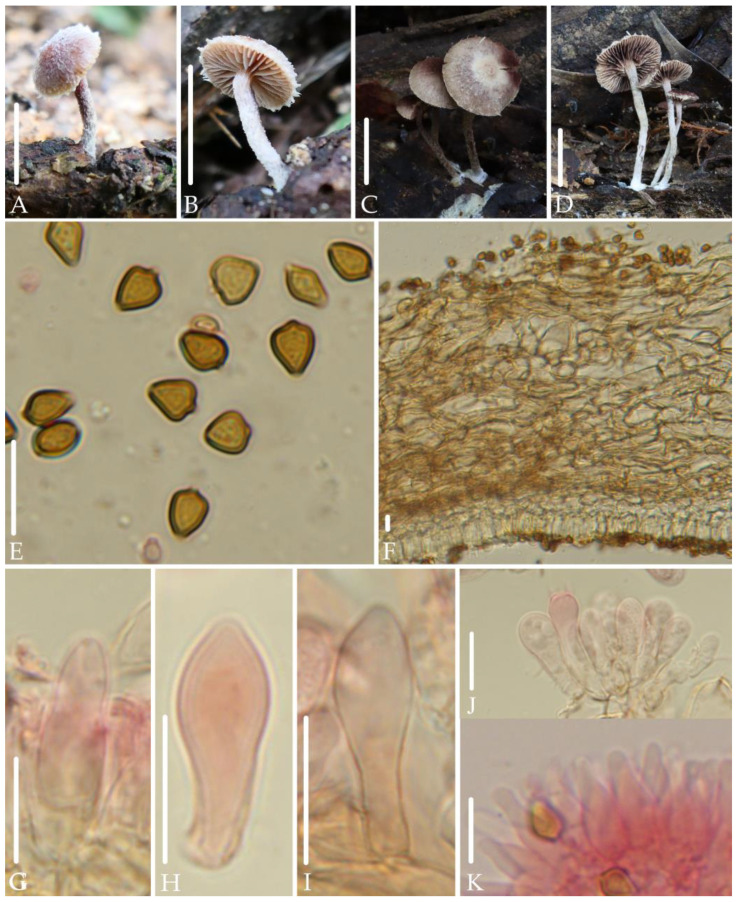
Morphological structures of *Deconica furfuracea.* (**A**–**D**) Basidiomata. (**E**) Spores. (**F**) Pileipellis. (**G**–**I**) Pleurocystidia. (**J**) Hymenium. (**K**) Cheilocystidia. Scale bars: (**A**–**D**) 10 mm, (**E**–**K**) 10 μm. All microstructures were observed in 5%KOH. Structures of (**G**–**K**) were stained with 1%Congo red.

*Deconica furfuracea* J.Q. Yan, S.N. Wang, and H. Zeng sp. nov. (Figure 3).

MycoBank: 855639

*Etymology*. “furfuracea” refer to its well-developed veil in early stage.

*Holotype*. China, Fujian Province, Wuyishan National Park, 8 June 2022, collected by Cheng-Feng Nie, Sheng-Nan Wang, HFJAU5181.

*Diagnosis*. *Deconica furfuracea* is mainly characterized by very small basidiomata; hygrophanous, dull red pileus; well-developed and evanescent veil; rhomboid to mitriform spores in face view, (5.0) 5.5–6.5 (7.5) × 4.5–5.2 (6.0) µm; thin-walled pleurocystidia that are small, rarely, fusiform to subclavate with subacute apex; fusiform to sublageniform and thin-walled cheilocystidia. It differs from *D. thailandensis* by lack of chrysocystidia.

*Description*. Basidiomata very small. Pileus 6.5–12 mm, convex to plano-convex, with or without an inconspicuous obtuse umbo in the center, hygrophanous, dull red (8C5–8C6), gradually paler toward the margin, grayish orange to brownish orange (6B6–6C6), becoming brownish orange (6B6–6C6) as drying. Veil well developed in early stage, white, fibrillose, evanescent. Context thin, 1.0 mm at the center. Lamellae 2.0 mm broad, adnate to decurrent, distant, unequal, light orange to grayish orange (6A5–6B5), edge white. Stipe 9.0–20 mm long, 1.0–2.0 mm thick, central, cylindric, equal, dull red (8C5–8C6), covered by abundant white fibrillose.

Spores (5.0) 5.5–6.5 (7.5) × 4.5–5.2 (6.0) µm, Q = (1.1) 1.2–1.5 (1.6), rhomboid to mitriform in face view, ellipsoid to elongated-ellipsoid in profile, 3.5–4.0 (4.5) µm broad, slightly thick-walled, smooth, brownish-yellow, germ pore distinct, 1.0–1.5 µm broad. Basidia (13) 15–21 × 4.4–6.7 µm, subclavate to cylindric, pale yellow to hyaline, 4-spored. Pleurocystidia 15–23 (26) × 5.3–7.7 µm, rarely, fusiform to subclavate, thin-walled, subacute at apex, hyaline. Cheilocystidia (9.0) 15–18 (20) × (4.3) 4.9–6.7 (7.8) µm, fusiform to sublageniform, rarely mucronate at apex, thin-walled, hyaline. Pileipellis a cutis, hyphae (4.0) 5.0–11 (12) µm broad, cylindric, slightly gelatinous, pale yellow to pale yellow-brown. Clamp connections present.

*Habitat.* Solitary to scattered on rotten wood in mixed coniferous and broad-leaved forests.

*Additional specimens examined*: China, Jiangxi Province, Jiangxi Agricultural University, 2 June 2019, collected by Jun-Qing Yan, HFJAU1250; Fujian Province, Wuyishan National Park, 26 June 2022, collected by Zhi-Heng Zeng, Hui Zeng, HFJAU3793; 8 June 2022, collected by Cheng-Feng Nie, Sheng-Nan Wang, HFJAU5181.

*Notes*. This species forms an independent branch in the phylogenetic tree, showing clear genetic differentiation from other known *Deconica* spp. Morphologically, the pleurocystidia of *D. furfuracea* in question are similar in size to the basidioles, and due to their rarity, they are easily overlooked and confused with basidioles. However, the apex of basidioles of this species are obtuse to broadly obtuse, while the apex of pleurocystidia are subacute. This phenomenon of confusion between pleurocystidia and basidioles is not unique within this genus. *D. thailandensis* (E. Horak, Guzmán & Desjardin) Ram.-Cruz & Guzmán also has pleurocystidia that are subclavate, thin-walled, and shorter than 25 µm in length. However, the presence of annulus and chrysocystidia can clearly distinguish *D. thailandensis* from *D. furfuracea* [31].

Among the known species of the *Deconica*, few have a similar combination of characteristics as *D. furfuracea*, that is, spores that are rhomboid to mitriform in face view, with a length concentrated between 5.5–6.5 μm, and have pleurocystidia. However, they can be clearly distinguished from *D. furfuracea*: *D. esperancensis* Ram.-Cruz & Glez.-Adam, *D. overeemii* (E. Horak & Desjardin) Desjardin & B.A. Perry, *D. umbrina* (E. Horak, Guzmán & Desjardin) Ram.-Cruz & Guzmán have chrysocystidia [1,17,31]; the pleurocystidia of *D. flocculosa* (Bas & Noordel.) Noordel. are ventricose-rostrate or lageniform (Bas and Noordeloos, 1996).

**Figure 4:**
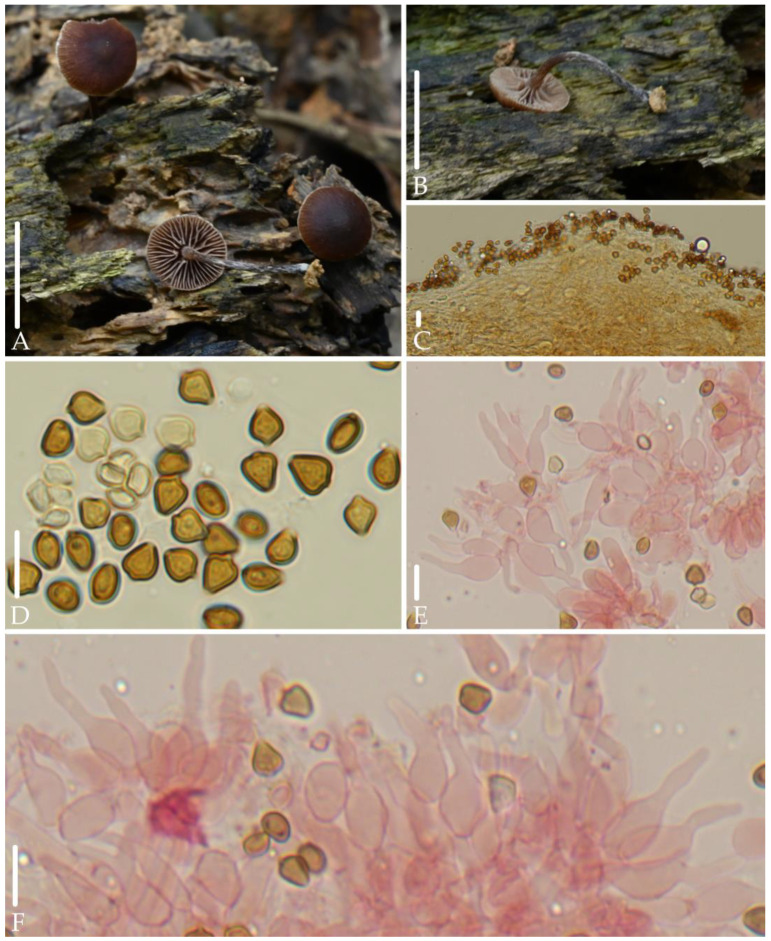
Morphological structures of *Deconica fuscobrunnea.* (**A**,**B**) Basidiomata. (**C**) Pileipellis. (**D**) Spores. (**E**,**F**) Cheilocystidia. Scale bars: (**A**,**B**) 10 mm, (**C**–**F**) 10 μm. All microstructures were observed in 5%KOH. Structures of (**E**,**F**) were stained with 1%Congo red.

*Deconica fuscobrunnea* J.Q. Yan, S.N. Wang, and H. Zeng sp. nov. (Figure 4).

MycoBank: 855640

*Etymology*. “fuscobrunnea” refer to its dark brown pileus.

*Holotype*. China, Fujian Province, Wuyishan National Park, 19 May 2022, collected by Jun-Qing Yan, Hu Zeng, Zhi-Heng Zheng, HFJAU3517.

*Diagnosis*. *Deconica fuscobrunnea* is mainly characterized by very small basidiomata; hygrophanous, dark brown pileus; decurrent and distant lamellae; rhomboid to mitriform spores in face view, 5.0–6.5 (7.0) × 4.5–5.3 (5.6) µm; lageniform cheilocystidia with long neck; ixocutis of pileipellis. It differs from *D. esperancensis* by lack of pleurocystidia.

*Description*. Basidiomata very small. Pileus 5.0–6.0 mm, plano-convex to plane, with unobviously obtuse umbo in the center, hygrophanous, dark brown (8F7–8F8) to reddish brown (8E7–8E8), gradually paler toward the margin, striate up to 1/2 from the margin. Veil only remains on the edge of the pileus, fibrillose, white, evanescent. Context thin, 1.0 mm at the center. Lamellae 1.0–1.5 mm broad, decurrent, distant, unequal, rarely forked, grayish orange (6B4–6C4), edge white. Stipe 9.0–20 mm long, 1.3–2.0 mm thick, central, cylindric, equal, reddish brown (8D5–8E5), gradually darkening toward the base, covered with white and evanescent fibrillose.

Spores 5.0–6.5 (7.0) × 4.5–5.3 (5.6) µm, Q = 1.0–1.3 (1.5), rhomboid to mitriform in face view, 3.6–4.3 (4.6) µm broad, ellipsoid to elongated-ellipsoid in profile, slightly thick-walled, smooth, brownish-yellow, germ pore distinct, 0.6–1.5 µm broad. Basidia 13–22 × 5.0–6.6 µm, subclavate to cylindric, pale yellow to hyaline, 4-spored. Pleurocystidia absent. Cheilocystidia 12–25 (28) × 4.0–8.0 (9.0) µm, lageniform, with long neck, rarely forked, thin-walled, pale yellow to hyaline; Pileipellis an ixocutis, hyphae 3.6–5.3 (6.4) µm broad, cylindric, gelatinous, pale yellow. Clamp connections present.

*Habitat.* Scattered on rotten wood in broad-leaved forests.

*Additional specimens examined*: China, Fujian Province, Wuyishan National Park, 19 May 2022, collected by Jun-Qing Yan, Hu Zeng, Zhi-Heng Zheng, HFJAU3517; 19 May 2023, collected by Jun-Qing Yan, Sheng-Nan Wang, HFJAU5706.

*Notes*. This species forms an independent branch and is grouped with *D. baylisiana* (E. Horak) J.A. Cooper, *D. esperancensis*, *D. milvispora* Ram.-Cruz & Matheny, *D. novae-zelandiae* (Guzmán & E. Horak) J.A. Coope and *D*. sp.1 from China. However, spores of *D. baylisiana*, *D. milvispora*, and *D. novae-zelandiae* are longer than 7.5 µm [1,32,33]; *D. esperancensis* has chrysocystidia [1]; *D.* sp.1 has chrysocystidia and spores that are ellipsoid to ovoid, rarely rhomboid in face view.

Morphologically, among the known species of the *Deconica*, only *D. deconicoides* (E. Horak, Guzmán & Desjardin) Guzmán and *D. xeroderma* share a similar combination of characteristics as *D. fuscobrunnea*, that is, spores that are rhomboid to mitriform in face view, with a length concentrated between 5.0–6.5 μm, and lacks pleurocystidia. However, the two former species do not have ixocutis of pileipellis, and their cheilocystidia are fusiform [34,35].

**Figure 5:**
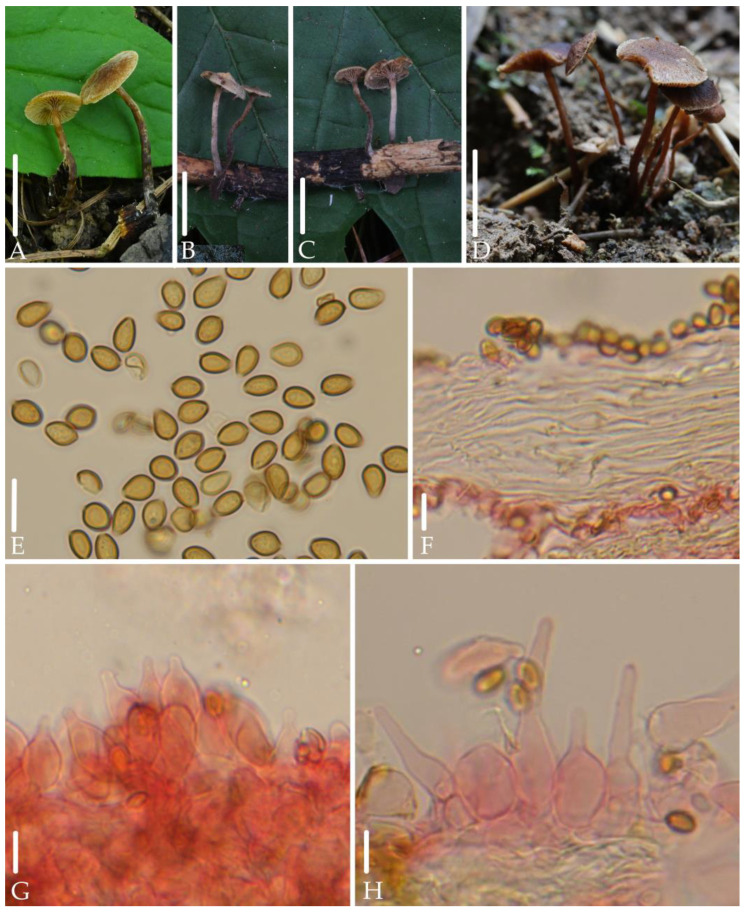
Morphological structures of *Deconica ovispora.* (**A**–**D**) Basidiomata. (**E**) Spores. (**F**) Pileipellis. (**G**,**H**) Cheilocystidia. Scale bars: (**A**–**D**) 10 mm, (**E**–**H**) 10 μm. All microstructures were observed in 5%KOH. Structures of (**F**–**H**) were stained with 1%Congo red.

*Deconica ovispora* J.Q. Yan, S.N. Wang, and H. Zeng sp. nov. (Figure 5).

MycoBank: 855641

*Etymology*. “ovispora” refer to its ovoid spores in face view.

*Holotype*. Hubei Province, Xingshan County, Yichang City, 2 July 2024, collected by Jun-Qing Yan, Bin-Rong Ke, HFJAU5476.

*Diagnosis*. *Deconica ovispora* is mainly characterized by very small basidiomata; hygrophanous, reddish brown to dark brown pileus; decurrent and distant lamellae; ovoid spores in face view, 6.0–7.0 × 4.0–4.5 µm; lageniform cheilocystidia with long to short neck, ixocutis of pileipellis. It differs from *D. xeroderma* by ixocutis of pileipellis and lageniform cheilocystidia.

*Description.* Basidiomata very small. Pileus 5.0–6.0 mm, plano-convex to plane, or umbonate to unobviously bullate in the center, hygrophanous, reddish brown to dark brown (8D7–8E7), gradually paler toward the margin, striate up to 1/2 from the margin, becoming reddish gray to dull red (8B2–8B3) as drying. Veil white, fibrillose, scattered, evanescent. Context thin, 1.0 mm at the center. Lamellae 0.5–1.0 mm broad, decurrent, distant, unequal, brownish (6C5–6D5), edge white. Stipe 10–20 mm long, 1.0–2.0 mm thick, central, cylindric, equal, brown (7E5–7E6), gradually darkening toward the base, covered with white and evanescent fibrillose.

Spores 6.0–7.0 × 4.0–4.5 µm, Q = 1.4–1.6 (1.7), ovoid in face view, ellipsoid to elongated-ellipsoid in profile 3.5–4.0 µm broad, slightly thick-walled, smooth, brownish-yellow, germ pore distinct, 0.8–1.3 µm broad. Basidia 14–24 × 4.5–6.0 µm, subclavate, hyaline, 4-spored. Pleurocystidia absent. Cheilocystidia 21–36 × 5.5–14µm, lageniform, with long to short neck, thin-walled, pale yellow to hyaline; Pileipellis an ixocutis, hyphae 2.5–4.5 µm broad, cylindric, subgelatinous, pale yellow. Clamp connections present.

Habitat. Solitary, scattered or gregarious on rotten wood or humus in broad-leaved forest and mixed coniferous and broad-leaved forests.

*Additional specimens examined*: China, Zhejiang Province, Qingyuan County, 6 July 2020, collected by Jun-Qing Yan, Sheng-Nan Wang, HFJAU1915; Hubei Province, Xingshan County, Yichang City, 2 July 2024, collected by Jun-Qing Yan, Bin-Rong Ke, HFJAU5429, HFJAU5476.

*Notes*. *Deconica ovispora* and the unknown species collected from the USA (ON774784, OP270521, MT622259) form independent branches, and their ITS similarity is over 99%, which also suggests that this species is distributed in the USA. In addition, this species groups together with *D. xeroderma* and an unknown sequence from China. However, *D. xeroderma* does not have ixocutis of pileipellis, and its cheilocystidia are fusiform [34].

Morphologically, among the known species of the *Deconica*, few have a similar combination of characteristics as *D. ovispora*, that is, spores that are ovoid in face view, with a length concentrated between 6.0–7.0 μm, and lack pleurocystidia. However, they can be clearly distinguished from *D. ovispora*: *D. micropora* (Noordel. & Verduin) Noordel. has spores that are up to 6.0 µm broad, with indistinct germ pores, and it is symbiotic with mosses [36]; *D. musacearum* (Singer) Cortez & P.S. Silva has cutis of pileipellis, and smaller cheilocystidia, measuring 16–17.5 × 4.0–5.5 µm [37]; *D. phillipsii* (Berk. & Broome) Noordel. has pleurotoid basidiomata and narrowly fusiform cheilocystidia [38].

## 4. Discussion

*Deconica* was one of the largest genera with an unsequenced generic type, and due to this limitation, the diversity of species within this genus may be underestimated. In Ramírez-Cruz’s study of 61 samples on a fairly global basis, there are as many as 18 undescribed species [1]. In this article, the publication of two new species, *D. ovispora* and *D. furfuracea*, addresses two undescribed species proposed by Ramírez. However, it also reveals the potential for more new species, such as *D*. sp.1 from China. Due to the quality of the specimens, we did not observe the cheilocystidia, but based on the other observed morphological features, *D*. sp.1 can be well distinguished from the known species of *Deconica*.

The confusion in species names in GenBank further complicates the recognition of species within this genus. For example, the branch formed by the new species *D. furfuracea* in the phylogenetic tree includes sequences from South Korea (MF437002), the United States (MK607529, FJ596920, FJ596921), which were previously named as *D. phyllogena* or its synonyms, and shares over 99% ITS similarity with them. However, it is well known that *D. phyllogena* has a viscid and smooth pileus, lacks pleurocystidia, spores that can be as wide as 6.5 μm, and cheilocystidia longer than 25 μm, which can be distinctly differentiated from *D. furfuracea* [39,40]. The photograph of the pileus with a well-developed veil corresponding to the sequence MK607529 further confirms that these sequences were incorrectly identified. Therefore, the diversity within the species of the *Deconica* deserves more attention.

The most recent infrageneric classification of *Deconica* was the work by Noordeloos, who divided *Deconica* into three sections: *Deconica* (with two subsections: *Deconica* and *Inquilinae*), *Melanotus*, and *Merdariae* [2]. However, the infrageneric classification only considered species from Europe and was not supported by molecular phylogenetic research [1].

The results of our phylogenetic analysis were somewhat consistent with previous studies by Ramírez-Cruz et al., showing that some clades were strongly supported but difficult to morphologically characterize [1]. The clade containing *D. citrispora* (E. Horak) J.A. Cooper and *D. neorhombispora* (Guzmán) P.S. Silva, Ram.-Cruz & Guzmán includes over 20 species and has a strongly supported (BI = 0.97, ML = 88). This clade, pointed out by Ramírez-Cruz et al., corresponds to *Psilocybe* sect. *Chrysocystidiatae* sensu Singer, and is predominantly composed of taxa with chrysocystidia [17]. Nevertheless, more than ten species including the three new species, *D. austrosinensis*, *D. furfuracea*, and *D. fuscobrunnea*, discovered in this study, all lack chrysocystidia. Therefore, the morphological characterization viewpoint of this clade is difficult to sustain. Fortunately, the species diversity of this genus may be much richer than we previously thought. As more species are discovered, we believe that morphologically synapomorphies will be better resolved in the phylogenetic tree.

## Data Availability

The sequences generated in this study are available in NCBI GenBank under the accession numbers shown in Table 1. The specimens studied in this study were deposited in the Herbarium of Fungi, Jiangxi Agricultural University (HFJAU). All alignments for phylogenetic analyses were deposited in TreeBASE (ID: TB2:S31681); the following links were available: http://purl.org/phylo/treebase/phylows/study/TB2:S31681?x-access-code=a06fbd515d1b8da177f23c6f45f2aaf2&format=html (accessed on 5 September 2024).
